# Allelic Diversity, Structural Analysis, and Genome-Wide Association Study (GWAS) for Yield and Related Traits Using Unexplored Common Bean (*Phaseolus vulgaris* L.) Germplasm From Western Himalayas

**DOI:** 10.3389/fgene.2020.609603

**Published:** 2021-01-28

**Authors:** Reyazul Rouf Mir, Neeraj Choudhary, Vanya Bawa, Sofora Jan, Bikram Singh, Mohd Ashraf Bhat, Rajneesh Paliwal, Ajay Kumar, Annapurna Chitikineni, Mahendar Thudi, Rajeev Kumar Varshney

**Affiliations:** ^1^Division of Genetics and Plant Breeding, Faculty of Agriculture, Sher-e-Kashmir University of Agricultural Sciences and Technology of Kashmir (SKUAST-K), Sopore, India; ^2^Division of Plant Breeding and Genetics, Sher-e-Kashmir University of Agricultural Sciences and Technology of Jammu, Jammu, India; ^3^The International Institute of Tropical Agriculture, Ibadan, Nigeria; ^4^Department of Plant Sciences, North Dakota State University, Fargo, ND, United States; ^5^Center of Excellence in Genomics and Systems Biology, International Crops Research Institute for the Semi-Arid Tropics, Hyderabad, India

**Keywords:** common bean, north-western Himalayas, allelic diversity, structural analysis, GWAS, QTLs/genes for yield traits

## Abstract

The north-western Indian Himalayas possesses vast diversity in common bean germplasm due to several years of natural adaptation and farmer’s selection. Systematic efforts have been made for the first time for the characterization and use of this huge diversity for the identification of genes/quantitative trait loci (QTLs) for yield and yield-contributing traits in common bean in India. A core set of 96 diverse common bean genotypes was characterized using 91 genome-wide genomic and genic simple sequence repeat (SSR) markers. The study of genetic diversity led to the identification of 691 alleles ranging from 2 to 21 with an average of 7.59 alleles/locus. The gene diversity (expected heterozygosity, *He*) varied from 0.31 to 0.93 with an average of 0.73. As expected, the genic SSR markers detected less allelic diversity than the random genomic SSR markers. The traditional clustering and Bayesian clustering (structural analysis) analyses led to a clear cut separation of a core set of 96 genotypes into two distinct groups based on their gene pools (Mesoamerican and Andean genotypes). Genome-wide association mapping for pods/plant, seeds/pod, seed weight, and yield/plant led to the identification of 39 significant marker–trait associations (MTAs) including 15 major, 15 stable, and 13 both major and stable MTAs. Out of 39 MTAs detected, 29 were new MTAs reported for the first time, whereas the remaining 10 MTAs were already identified in earlier studies and therefore declared as validation of earlier results. A set of seven markers was such, which were found to be associated with multiple (two to four) different traits. The important MTAs will be used for common bean molecular breeding programs worldwide for enhancing common bean yield.

## Introduction

Common bean (*Phaseolus vulgaris* L.) is one of the most important diploid grain legume crops (2*n* = 2*x* = 22) with a small genome size of 587 Mbp (Broughton et al., 2003). It is the major source of calories and proteins for the people in developing countries of the world (FAO^1^). Common bean is one of the most ancient crops of the Americas (Broughton et al., 2003; McClean et al., 2004) and possesses two important already diverged gene pool species: the Mesoamerican and Andean gene pool species. The Mesoamerican gene pool species is distributed from northern Mexico to Colombia, whereas the Andean gene pool species is distributed from southern Peru to north-western Argentina (Kwak and Gepts, 2009). The presence of two gene pools in common bean raises the following questions during common bean germplasm evaluation and characterization: (i) the relationship between the germplasm from two gene pools, (ii) the diversity/variation present within and between these gene pools, (iii) the quantitative differences in genetic diversity, and (iv) the levels of linkage disequilibrium (Kwak and Gepts, 2009). The characterization of genetic diversity is one of the most important subject areas of crop research. The characterized crop germplasm forms the basis of crop improvement programs and the development of genetic resources, such as mapping populations and core collections, for the genetic dissection of important traits through quantitative trait locus (QTL) mapping and genome-wide association mapping approaches (McClean et al., 2012).

A huge unexplored diversity has been observed in common bean germplasm in Jammu and Kashmir: a north-western Himalayan region in India and this region is famous for producing high-quality beans. The common bean germplasm from the area have different market classes, plant types, seed quality traits, and agro-ecological adaptation (Choudhary et al., 2018a,b). Keeping in view the diversity of common bean in this region, it will not be un-wise to call this area as “secondary center of diversity” for common bean. The huge diversity that is available in the common bean germplasm from western Himalayas of India is perhaps due to the differential adaptive evolutionary process that is happening continuously over the last several hundred years since their introduction in western Himalayas by travelers from Portugal, England, Holland, France, China, and Pakistan (Rana et al., 2015; Choudhary et al., 2018b). The extent of genetic diversity and the origin of common bean in the Jammu and Kashmir region were recently characterized using *Phaseolin locus* (*Phs*) assays and sequencing of internal transcribed spacer (ITS) region (Choudhary et al., 2018b). Out of a set of 428 common bean lines, a diverse subset of 96 lines was selected based on cluster analysis using few qualitative traits and site of collection. The core set of 96 lines comprised 54 local landraces from 11 hotspots of the Himalayan region of Jammu and Kashmir and 42 exotic lines from 11 different countries. The phaseolin patterns of these 96 lines revealed the presence of lines with “S”-type phaseolin and “T”-type phaseolin patterns. The common bean germplasm from the Kashmir region possess both S- and T-type phaseolins, whereas the germplasm from the Jammu region possess only S-type phaseolins. Few earlier studies have also attempted to characterize this huge diversity of common bean in north-western Himalayas using morphological traits (Sofi et al., 2014; Saba et al., 2016) and less reliable random amplification of polymorphic DNA (RAPD) markers or only limited simple sequence repeat (SSR) markers (Zargar et al., 2016).

Different genomics tools and molecular techniques now offer much better understanding to assess the ability of crop genetic diversity. SSR markers are considered suitable for assessing genetic variation and allele mining because they are highly informative (Powell et al., 1996; Gupta et al., 2002; Mir and Varshney, 2013; Mir et al., 2013). Their advantages for diversity studies also include uniform genome coverage, high levels of polymorphism, co-dominance, and an easy-to-implement, specific polymerase chain reaction (PCR)-based assay (Pejic et al., 1998; Gupta et al., 2002; Mir and Varshney, 2013; Mir et al., 2013). While going through the literature in common bean, it was noticed that molecular markers have played an important role in the characterization and assessment of genetic diversity of landraces and farmers varieties (Blair et al., 2006; Asfaw et al., 2009; Angioi et al., 2010; Sharma et al., 2013; Okii et al., 2014; Buah et al., 2017). The restriction fragment length polymorphism (RFLP) markers were used as the first molecular marker system for the study of genetic diversity (Becerra-Velasquez and Gepts, 1994). The amplified fragment length polymorphism (AFLP) markers were used to study wild beans germplasm (Tohme et al., 1996), diversity and origin studies of Andean local landraces (Beebe et al., 2001), and DNA fingerprinting studies to characterize yellow beans from both gene pools (Pallottini et al., 2004). The RAPD markers were mainly used to study genetic diversity and population structure among common bean germplasm and landraces (Beebe et al., 2000; Razvi et al., 2013; Bukhari et al., 2015). SSR markers have been widely used in genetic diversity and population structure studies (Blair et al., 2006, 2009; Benchimol et al., 2007; Khaidizar et al., 2012; Okii et al., 2014; Fisseha et al., 2016). However, it is important to mention here that common bean population studies with SSR markers have been performed using only a small number of landraces or breeding lines or they have focused on certain geographic regions only (Metais et al., 2002; Blair et al., 2006; Dıaz and Blair, 2006). However, a systematic effort to characterize this huge diversity of Himalayan beans using molecular markers is still not available, although extremely useful for bean improvement.

Therefore, the present study was conducted to better understand the genetic diversity and population structure available in Himalayan bean germplasm using SSR markers. Efforts were also made to compare the genetic diversity revealed by genic and random SSR markers. Genome-wide association studies (GWASs) were conducted to identify molecular markers associated with yield and yield-contributing traits using precise and accurate genome-wide SSR marker data and trait data on yield and yield-contributing traits collected over 2 years. The knowledge and genetic/genomics resources (candidate genotypes for yield and related traits, associated markers, validated markers, and major/stable genes/QTLs) generated/developed in this study will be invaluable to the bean breeding programs aimed at improving yield and related traits in common bean throughout the world.

## Materials and Methods

### Plant Materials

The present study comprised a core set of 96 diverse common bean genotypes, including 54 local landraces from 11 hotspots of Jammu and Kashmir and 42 exotic lines belonging to 11 different countries. The 96 lines include 51 Andean types with “T”-type phaseolin and 45 Mesoamerican types having “S”-type phaseolin. Among 54 local landraces, 32 lines are Andean type and 22 lines are Mesoamerican type. Among 42 exotic lines, 19 are Andean type and 23 are Mesoamerican type (Supplementary Table 1). The diverse 96 lines have been carefully selected from a set of 428 lines based on the evaluation of qualitative data, such as seed color, seed shape, flower color, and distribution in different regions in Jammu and Kashmir, India and other 11 different countries (for more details about germplasm and selection criteria, see Choudhary et al., 2018b). In short, both quality data and information about their collection sites were kept into consideration while selecting the diverse set of 96 lines. The quality data of 428 lines were used in clustering, and a dendrogram was prepared. The dendrogram in addition to landrace collection site information was used for the selection of the final set of 96 lines (Choudhary et al., 2018b). The local landraces were collected from different common bean growing regions of Jammu and Kashmir, and exotic lines were procured from the National Bureau of Plant Genetic Resources (NBPGR), Shimla, Himachal Pradesh, India.

### Trait Phenotyping and Analysis of Data

The diverse set of 96 lines was phenotyped for four important yield-related traits including pods per plant, seeds per pod, 100-seed weight (g), and yield per plant (g). The data on these traits were recorded at two important common bean growing regions in Jammu and Kashmir, i.e., at Bhaderwah (32.980033°N 75.713706°E) located at an elevation of 5292 ft and at SKUAST-Jammu, Chatha-Jammu, India (32.73°N 74.87°E) located at an elevation of 1000 ft. The 96 genotypes were evaluated in an Augmented Block Design (ABD) that consisted of six blocks, each containing 16 genotypes and three local checks allotted to each block randomly. The plots were kept free from weeds, diseases, and pests throughout the cropping cycle. Standard agronomic practices were followed for normal crop growth during both years. Five plants in each genotype were selected for recording the data, and the mean data from two locations were used in statistical analysis. The mean data were analyzed to estimate variability parameters, such as phenotypic coefficient of variation (PCV), genotypic coefficient of variation (GCV), heritability, genetic advance, and correlation coefficient, using the software program “Windostat ver 9.2^2^” developed by Indostat services, Hyderabad, India^3^. The data on these four traits for both the locations were also utilized to identify significant marker–trait associations (MTAs) in GWAS using different software programs.

### Genomic DNA Extraction

The genomic DNA of the collection of bean genotypes was extracted using the Qiagen DNeasy DNA extraction kit following standard protocols. More details about checking of quantity and quality are available elsewhere (Choudhary et al., 2018a).

### Selection of SSR Markers

A set of markers (91 SSR markers) selected for this work was done based on several parameters and criteria and included: (i) high polymorphic information content (PIC) values (>0.6), (ii) maximum number of alleles detected in earlier studies, (iii) genic SSR vs. random genomic SSR, and (iv) uniform distribution on all the 11 linkage groups. The details of these 91 SSR markers are also available elsewhere (see Supplementary Tables 2, 3). Out of these 91 selected markers, 32 were either genic markers associated with different traits or EST-derived SSR markers (Supplementary Table 3). The markers and their primer sequences once selected were synthesized on contract from Sigma–Aldrich, Bangalore, India.

### PCR and SSR Marker Genotyping

The genotyping of 45 SSR markers was done in the Molecular Breeding Laboratory of the Division of Genetics and Plant Breeding, SKUAST-Jammu, Chatha, Jammu using polyacrylamide gel electrophoresis (PAGE) systems (High-throughput Dual Gel Vertical Electrophoresis System) by Peqlab/CBS Scientific, United States, followed by silver staining before recording the data. For PAGE, the PCR amplifications were done in 10 μl reaction volume using 20 ng of DNA template, 5.0 pmol forward reverse primers, 2.5 mM of each dNTPs, 1 × buffer, 2.0 mM MgCl_2_, 10 mM Tris–HCl, 50 mM KCl, and 1.0 U of *Taq* polymerase (Sigma/HiMedia). The thermal cycler (Peqlab) was programmed as follows: initial denaturation at 95°C for 5 min, 40 cycles of 94°C for 1 min of denaturation, 50–60°C for 1 min of annealing temperature, 72°C for 1 min, and final extension at 72°C for 8 min. The resulting PCR products were run in 10% PAGE to score the allele polymorphism of various markers.

In addition, the genotyping was also done for 46 SSR markers using an ABI 3730 automatic DNA Sequencer Genotyping Platform (Applied Biosystems, Foster City, CA, United States) at the Centre of Excellence in Genomics and Systems Biology (CEGSB), ICRISAT, Hyderabad, Telangana, India. The genotyping involves PCR amplifications of SSR loci using a thermal cycler (GeneAmp PCR System 9700; Applied Biosystems, Foster City, CA, United States), followed by amplification on 1.2% agarose gel for confirming PCR amplification. Separation of amplified products was done using capillary electrophoresis and GeneMapper software version 4.0 (Applied Biosystems, Foster City, CA, United States).

### SSR Marker Data Analysis

Several parameters of genetic diversity including the most important PIC value and the number of alleles/locus were used to assess the extent of genetic diversity available in the common bean core set. The GenAlEx software program (Peakall and Smouse, 2006) was used to calculate genetic diversity parameters, such as genetic distance, number of alleles, number of effective alleles, number of private alleles, number of common alleles, observed heterozygosity, and expected heterozygosity. The diversity parameters were calculated separately for random genomic SSR markers and genic SSR markers, as well as together on the whole population. The analysis was repeated separately by classifying the core set of 96 lines into exotic vs. local landraces and Mesoamerican vs. Andean gene pool landraces. The PIC value for each SSR was calculated manually using Microsoft Excel following Botstein et al. (1980). DARwin version 5.0 was used to calculate pair-wise genetic distances and to construct the dissimilarity matrix (Perrier et al., 2003). The dissimilarity matrix thus obtained was subjected to cluster analysis using the unweighted neighbor-joining (UNJ) method (Gascuel, 1997), followed by bootstrap analysis with 1000 permutations to obtain a dendrogram (Perrier et al., 2003; Mir et al., 2012a).

#### Analysis of Molecular Variance (AMOVA)

To test the genetic variation within and between cultivars of exotic and local landraces, analysis of molecular variance (AMOVA) was carried out using the software program GenAlEx (Peakall and Smouse, 2006).

#### Population Structure Analysis

Population structural analysis, which is a model-based clustering, was done to find out the number of subpopulations in our common bean population of 96 lines, using the software program STRUCTURE version 2.3.4 (Pritchard et al., 2000). We tested the number of subpopulations (K) from 1 to 10, and each was repeated three times. For each run, burn-in was set at 100,000, iteration was set at 200,000, and a model without admixture and correlated allele frequencies was used. The run with maximum likelihood was used to assign our 96 common bean lines into subpopulations. This assignment obtained through maximum-likelihood approach was further confirmed by a modified Delta-K (ΔK) method, which provides the real number of clusters/subpopulations (Evanno et al., 2005). Within a subpopulation, the genotypes with affiliation probabilities (inferred ancestry) ≥ 80% were assigned to a distinct subpopulation, and those with < 80% were treated as admixture, i.e., these genotypes seem to have a mixed ancestry from parents belonging to different gene pools or geographical origin (Mir et al., 2012b).

### MTAs for Yield and Yield-Contributing Traits

Association mapping was conducted for the identification of significant MTAs for yield and yield-contributing traits. The trait data on 100-seed weight, pods per plant, seeds per pod, and yield per plant for two locations along with SSR marker data were used in the software program TASSEL 3.0^4^ to identify significant MTAs. The analysis of MTAs was done using two different models including general linear model (GLM) based on the Q-matrix derived from the STRUCTURE software and mixed linear model (MLM) based on both the Q-matrix and the kinship matrix (K-matrix) derived from the marker data using the TASSEL software program (for details, see Choudhary et al., 2018a). The significance of MTAs was described in terms of *P*-value (*P* ≤ 0.05 for significant markers). The Manhattan plot and quantile–quantile (QQ) plot were also prepared using the software program TASSEL.

## Results

### Trait Variability for Four Yield-Contributing Traits

Yield-contributing traits, such as the number of pods/plant, the number of seeds/pod, 100-seed weight, and grain yield/plant, are important target traits in common bean breeding programs worldwide. During the present study, the analysis of these four important traits in a core set of 96 lines revealed a broad spectrum of variation as indicated by the wide range and high PCV and GCV values. The GCV values were the highest for yield per plant (59.56), followed by 100-seed weight (38.86) and pods per plant (38.12), whereas a lower value was recorded for seeds per pod (16.97). GCV values were lower than PCV values for all traits indicating a significant influence of environment on these traits, underlining the need to test the stability of performance across a range of environments (Table 1). A similar trend was observed for broad sense heritability and genetic advance. The highest expected genetic advance (measure of genetic gain while exercising selection) was observed for yield (110.47%), followed by 100-seed weight and pods per plant, whereas the lowest value was recorded for seeds per pod (26.82%) (Table 1). The parameters including PCV, GCV, heritability, and expected genetic gain are of paramount importance as they define the limits of genetic gain that can be achieved through selection. In the present study, all the component traits were significantly correlated with grain yield (Table 1).

**TABLE 1:** Trait variability for yield and yield-contributing traits in pooled data over different environments.

Trait	Mean ± SE	Range	PCV	GCV	Heritability	Genetic advance as% of mean	Correlation with yield per plant
Pods per plant	9.61 ± 0.83	5.10–18.59	40.28	38.12	85.32	67.01	0.596**
Seeds per pod	5.07 ± 0.27	3.23–7.31	18.80	16.97	76.52	26.82	0.516**
100-seed weight (g)	32.05 ± 0.29	11.57–65.84	38.90	38.86	98.83	80.56	0.516**
Yield per plant (g)	15.47 ± 1.77	3.61–69.24	61.04	59.56	80.02	110.47	–

PCV, phenotypic coefficient of variation; GCV, genotypic coefficient of variation; SE, standard error. **indicates significance at p = 0.01.

#### Analysis of Variance (ANOVA)

The analysis of variance (ANOVA) of the field experiment of bean germplasm at two locations (Jammu and Bhaderwah during *Rabi* 2014–15 and *Kharif* 2015) was conducted for four quantitative traits including pods per plant, seeds per pod, 100-seed weight, and yield per plant, and the results of the mean sum of squares (MSS) were calculated separately for both locations. Non-significant difference was found among all four traits at Jammu location (Table 2), but among the genotypes, all four traits exhibited significant differences (*P* = 0.01). Similarly, for data recorded at Bhaderwah, the differences were non-significant for replication, but significant among genotypes (*P* = 0.01).

**TABLE 2:** Analysis of variance for morphological traits of 96 common bean lines at two testing locations (SKUAST-Chatha, Jammu and Bhaderwah, Jammu).

Source of variation	df	Location	Mean squares			
			Pods per plant	Seeds per pod	100-seed weight (g)	Yield per plant (g)
Replications	2	SKUAST-Chatha	0.46	0.44	0.29	1.11
		Bhaderwah	3.32	0.19	0.74	7.02
Genotypes	95	SKUAST-Chatha	36.93**	2.09**	448.3**	253.00**
		Bhaderwah	45.07**	2.51**	483.57**	276.12**
Error	190	SKUAST-Chatha	2.05	0.18	0.18	9.88
		Bhaderwah	2.02	0.25	0.36	8.90
Total	287	SKUAST-Chatha	15.99	-0.08	129.00	279.83
		Bhaderwah	148.63	-0.52	356.84	1937.36

*Significant at 5% level. **Significant at 1% level.

#### Trait Correlations

The correlation analysis showed a significant positive correlation between seeds per pod with pods per plant. Yield per plant showed positive and highly significant correlations with three other yield component traits, *viz*., pods per plant, seeds per pod, and 100-seed weight at both locations. However, 100-seed weight has a significant negative correlation with pods per plant at both locations (for more details, see Supplementary Table 4).

### Allelic Diversity

Among all the 91 SSR markers tested on a set of 96 common bean lines, only one SSR marker “BMd44” was found to be monomorphic. The remaining 90 SSR markers detected multiple alleles in 96 genotypes. A total of 691 alleles were detected in all the 96 genotypes by 90 polymorphic SSR markers. The number of alleles detected varied from 2 for SSR marker Bmr205 to 21 for SSR marker BM187, with an average of 7.59 alleles/locus (Supplementary Table 2). The number of alleles with a frequency ≥ 5% was 5.31, and the number of effective alleles was 4.86. Similarly, gene diversity (expected heterozygosity, *He*) varied from 0.31 to 0.93 with an average of 0.73 (Table 3). The lowest *He* was recorded for SSR marker GATS54 and the highest for SSR marker BM187.

**TABLE 3:** Summary of different allelic diversity attributes of all 91 SSR markers, genic markers, and random SSR markers in a single population of 96 common bean lines and in two subpopulation populations (indigenous vs. exotic and Mesoamerican vs. Andean populations).

Allelic diversity attribute	Single population of 96 lines			Exotic	Indigenous	Exotic	Indigenous	Exotic	Indigenous	Mesoamerican	Andean
						
	All 91 SSR markers	Random 59 markers	Genic 32 markers	All 91 markers		Random 59 markers		Genic 32 markers		All 91 markers	
**Na**	7.593	7.966	6.906	6.824	6.703	7.220	7.169	6.094	5.844	6.297	6.341
**Na freq. ≥5%**	5.308	5.797	4.406	4.912	4.967	5.220	5.458	4.344	4.063	4.571	4.418
**Ne**	4.858	5.336	3.975	4.559	4.426	4.835	4.872	4.050	3.604	4.159	3.950
**I**	1.611	1.713	1.423	1.550	1.530	1.634	1.633	1.395	1.338	1.413	1.378
**No. of private alleles**	7.593	7.966	6.906	0.890	0.769	0.797	0.746	1.063	0.813	1.253	1.297
***He***	0.734	0.766	0.676	0.722	0.715	0.751	0.746	0.670	0.658	0.668	0.648
**UHe**	0.738	0.770	0.680	0.732	0.722	0.760	0.753	0.678	0.665	0.676	0.655

*Na, no. of alleles; Ne, no. of effective alleles; I, information index; *He*, expected heterozygosity; UHe, unbiased heterozygosity.*

### Allelic Diversity of Local vs. Exotic Beans

**FIGURE 1:**
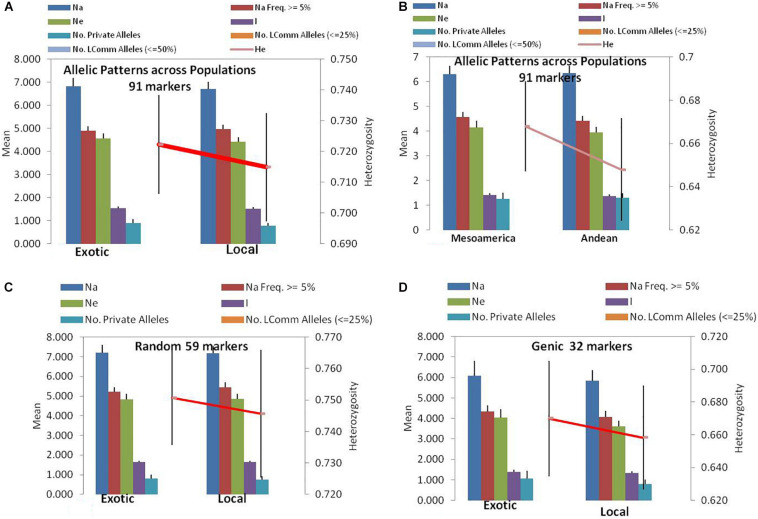
Allelic patterns in common bean by SSR markers during the present study: **(A)** Allelic pattern in exotic and local landraces by all 91 SSR markers. **(B)** Allelic pattern in Mesoamerican and Andean populations by all 91 markers. **(C)** Allelic pattern in exotic and local landraces by only random markers. **(D)** Allelic pattern in exotic and local landraces by only genic SSR markers. The red lines indicate the trend of change in diversity from one population/group to another population/group.

Among the 91 SSR markers tested on 54 local and 42 exotic common bean genotypes, we observed a total of 621 alleles in exotic germplasm and 610 alleles in local common bean germplasm. The number of alleles in exotic bean germplasm varied from 2 to 17 with an average of 6.82 alleles/locus. Similarly, the number of alleles in local bean germplasm varied from 2 to 16 with an average of 6.7 alleles/locus. The numbers of alleles with a frequency ≥ 5% were 4.92 for exotic and 4.97 for local lines. The numbers of effective alleles were 4.56 and 4.43, respectively, for exotic and local beans (Figure 1A). The number of private alleles in exotic beans was 81 against 70 in local common bean landraces with an average of 0.89 in exotic and 0.77 in local beans. The total number of common alleles between the two groups was 540 with an average of 5.94 alleles. Therefore, a set of 81 alleles was present exclusively in exotic beans, and 70 were present exclusively in local germplasm. While comparing gene diversity between the two groups, it was noticed that it does not differ much as the average *He* in exotic beans was 0.73 against 0.72 in local beans (Table 3 and Figure 1A).

### Allelic Diversity of Mesoamerican vs. Andean Beans

Among the 96 lines of core set, 51 lines belong to Andean types with “T”-type phaseolin, and the remaining 45 lines were of Mesoamerican type having “S”-type phaseolin. The 91 SSR markers tested during the present study detected 573 alleles in Mesoamerican beans (average: 6.30, range: 2–16) and 577 in Andean beans (average: 6.35, range: 2–15). The average private allele in Mesoamerican beans was 1.25 against 1.29 in Andean beans. We also observed that the average *He* in Mesoamerican beans was 0.67 against 0.65 in Andean beans. The Nei’s genetic distance between the two populations was found to be 0.61, and the genetic differentiation (pair-wise population Fst) between these two populations was found to be 0.116 (Table 3 and Figure 1B).

### Allelic Diversity by Genomic SSR Markers vs. Genic SSR Markers

The 59 polymorphic random genomic SSR markers detected 470 alleles with an average of 7.97 alleles, whereas 31 genic SSR markers detected a total of 221 alleles with an average of 6.90 alleles. The numbers of effective alleles detected were 5.34 and 3.98, respectively, by random and genic markers. While analyzing the data separately for exotic vs. local bean germplasm, it was observed that random markers detected 7.23 alleles in exotic and 7.17 alleles in local beans. The genic markers detected 6.1 alleles in exotic and 5.9 alleles in local beans (Table 3 and Figures 1C,D).

The number of private and common alleles detected was also compared between the random and genic SSR markers. The random SSR markers detected 47 (0.80 average) private alleles in exotic beans vs. 44 (0.75 average) in local beans. The genic SSR markers on the other hand detected 34 (1.1 average) private alleles in exotic vs. 26 (0.82 average) in local beans (Table 3). The total number of common alleles between exotic and local beans detected by random markers was 379 against only 161 by genic SSR markers.

The gene diversity (*He*) detected by random SSR markers was 0.77 and that of genic SSR markers was 0.68. While comparing the same separately for exotic and local beans, it was observed that random markers detected 0.75 in exotic and 0.75 in local beans. The genic markers on the other hand detected 0.68 in exotic and 0.66 in local beans (Table 3).

### Cluster Analysis

**FIGURE 2:**
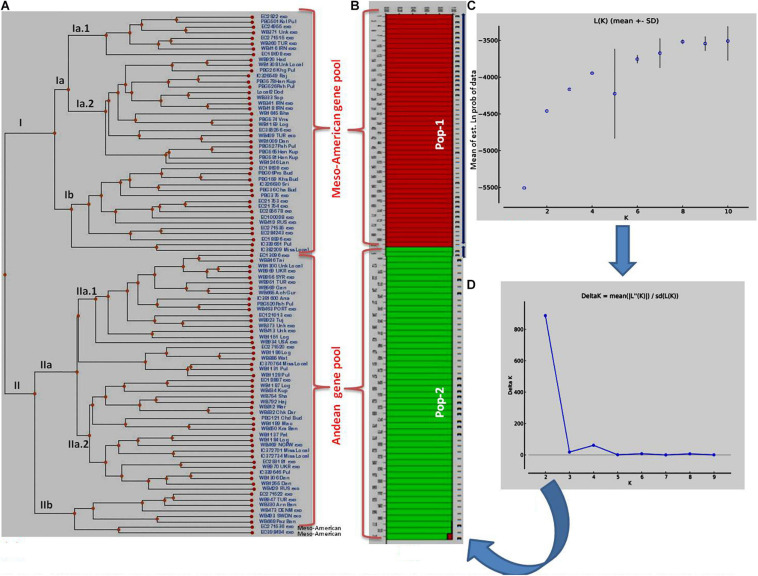
Clustering of 96 common bean genotypes: **(A)** Traditional UPGMA hierarchical clustering where 96 genotypes have been clustered into two groups (Mesoamerican vs. Andean types). **(B)** Bayesian clustering of 96 genotypes in the form of structure plots where two sub-populations could be easily distinguished from each other. The red plots show Mesoamerican sub-population, while green plots show Andean sub-populations. **(C)** Plot of Ln (K) values of different sub-populations from 1 to 10. **(D)** Rate of change of Ln (K) from sub-population 1 to 10 based on Delta-K method.

**FIGURE 3:**
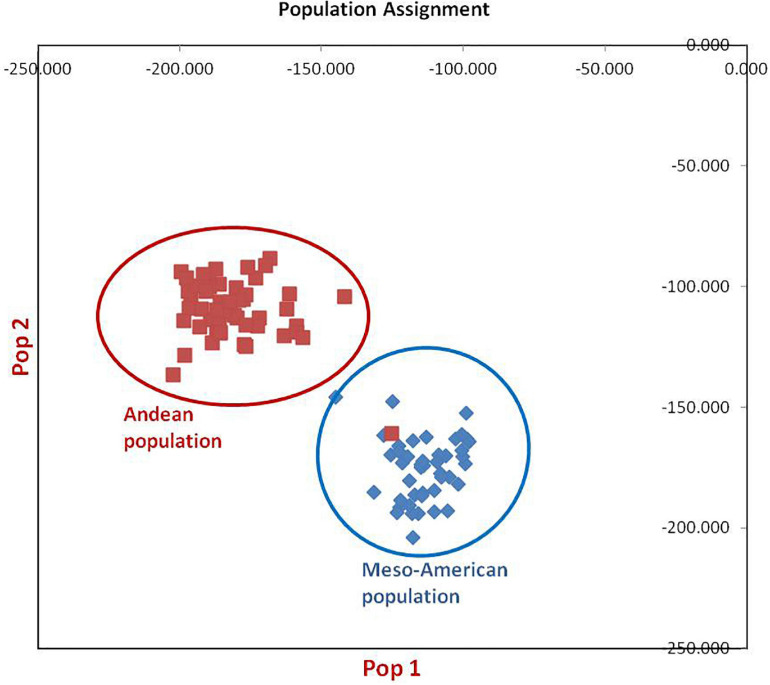
Population assignment of 96 common bean lines into two sub-populations. Population#1 possesses all individuals of Mesoamerican gene pool except one genotype from Andean population, while population#2 possesses all genotypes of Andean gene pool.

The clustering and construction of dendrogram based on 91 SSR markers led to the clustering/distribution of all the 96 lines into two main clusters (cluster I and cluster II). Cluster I was further divided into two sub-clusters (cluster Ia and cluster Ib). Sub-cluster Ia could be further divided into two sub-clusters, i.e., Ia.1 and Ia.2. The main cluster II could be divided into two sub-clusters, i.e., IIa and IIb. Sub-cluster IIa was further divided into two sub-clusters, i.e., IIa.1 and IIb.2 (Figures 2A–D). The exotic common bean lines from different countries other than India and indigenous local landraces collected from different hotspots of Jammu and Kashmir clustered together, and there was no clear-cut separation/clustering of local bean landraces from the exotic bean germplasm (ESM Figure 1). However, there was clear-cut clustering and assignment of Mesoamerican and Andean lines. All the Mesoamerican lines were clustered in cluster I except two lines (EC-271535 and EC-398494), which were clustered with Andean lines in sub-cluster IIb. On the other hand, all Andean gene pool lines were clustered separately in sub-cluster II (Figure 2A and Supplementary Table 1). Similar distinct clustering of the Mesoamerican and Andean gene pool lines except one line (WB1189) from the Andean gene pool got clustered with the Mesoamerican gene pool was also obtained by population assignment using GenAlEx ver 6.0 (Figure 3).

### Structural Analysis

The structural analysis using marker data led to the identification of two (K = 2) genetically distinct subpopulations (although 2–10 subpopulations were tested) in 96 diverse bean lines (Figure 2B). Initially, based on Mean LnP(K), the number of subpopulations could not be predicted since the probability values kept on increasing steadily up to K = 4 and then decreased at K = 5 and then again started increasing up to K = 9 before again started decreasing at K = 10 (Figure 2C and Supplementary Table 5). Thus, there was no clear trend emerging about the possible number of subpopulations using LnP(K) values. Therefore, the ΔK method by Evanno et al. (2005) was used to infer the correct number of subpopulations in our population of 96 common bean lines. The ΔK method takes the rate of change of the mean probability values (LnP) of each subpopulation into consideration. As per this method, the rate of change was maximum (1,615.97) at K = 2 (Figure 2D and Supplementary Table 5); therefore, we consider two subpopulations in our sample/population of 96 common bean lines (Figure 2B). Both these subpopulations possess equal 48 genotypes each. Subpopulation #1 contains 25 exotic lines and 23 local lines, whereas in subpopulation #2, the number of exotic lines was 17, and the number of local lines was 31. There was no clear trend of the distribution of local (indigenous lines) vs. exotic lines in structural plot (Figure 2), but the distribution was largely based on gene pool/phaseolin patterns. Subpopulation #1 possesses 41 individuals from the Mesoamerican gene pool possessing “S”-type phaseolin, and the remaining seven belong to the Andean gene pool with “T”-type phaseolin. On the other hand, subpopulation #2 possesses 44 individuals from the Andean gene pool possessing “T”-type phaseolin, and the remaining four individuals belonging to the Mesoamerican gene pool with “S”-type phaseolin. Further, all the lines in these two subpopulations possess an affiliation probability of >80%, and therefore no line has been declared as admixture between two subpopulations (Supplementary Table 1 for a structural matrix).

Average distances (expected heterozygosity) between individuals within clusters/subpopulations were also calculated using the software program STRUCTURE, and the analysis revealed that expected heterozygosity is more in the first subpopulation (0.6132) “Mesoamerican gene pool” than in the second subpopulation (0.5543) “Andean gene pool.” The allele-frequency divergence among populations (net nucleotide distance), computed using point estimates of P using the software program STRUCTURE, showed a distance of 0.2119 between the two subpopulations.

### Analysis of Molecular Variance

Analysis of molecular variance was conducted to test the existence of genetic structure among populations (bean accessions from Jammu and Kashmir vs. exotic beans from different countries), as well as among and within individuals. This analysis showed that the differences among the two bean populations (indigenous vs. exotic) were significant and explained 2.0% of the total genetic variance (Table 4). However, for the whole population, the major source of variance was among individuals and not within individuals (97 vs. 1%), reflecting the predominant self-pollinating reproductive system of the bean.

**TABLE 4:** Analysis of molecular variance (AMOVA) for the partitioning of microsatellite diversity.

Source	df	SS	MS	Est. var.	% Variation
Among pops	1	1,085,721.069	1,085,721.069	5698.423	2%
Among indiv	94	51,438,685.931	547,220.063	272,155.235	97%
Within indiv	96	279,321.000	2909.594	2909.594	1%
Total	191	52,803,728.000		280,763.252	100%

*SS, sum of squares; MS, mean squares.*

### Discovery of Important QTLs/Genes for Yield and Yield-Contributing Traits

**FIGURE 4:**
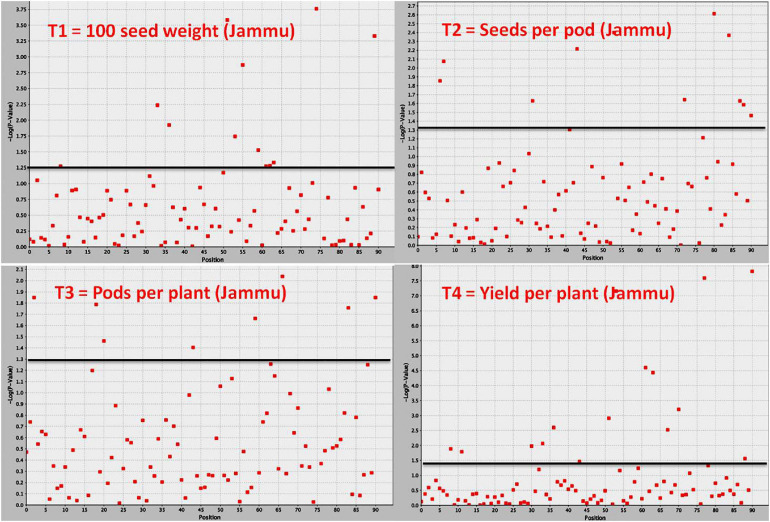
Manhattan plot showing significant MTAs identified using software program TASSEL for yield and yield-contributing traits in common bean. The MTAs have been identified using trait data of SKUAST-Jammu location, and significant MTAs for four traits are depicted above threshold lines.

**FIGURE 5:**
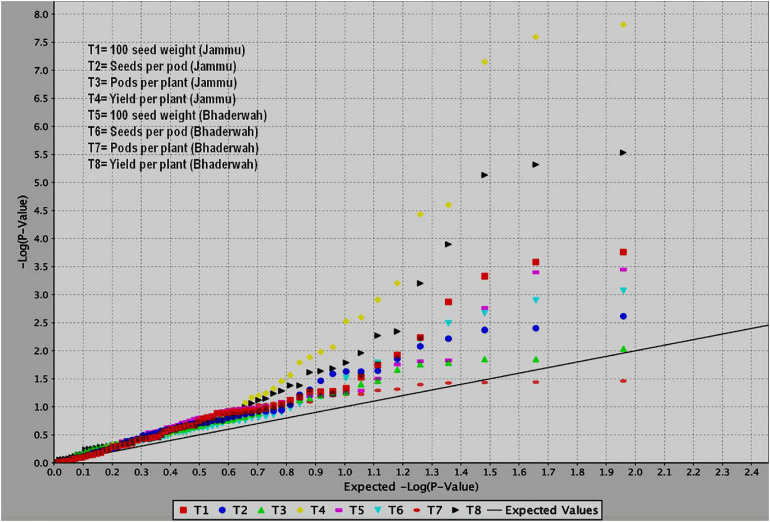
QQ plots obtained during study of marker–trait associations for yield and yield-contributing traits in common bean. The figure shows QQ plots for all the four traits in two different environments (T1–T4 at SKUAST Jammu and T5–T8 at Bhaderwah Jammu).

Association mapping identified a total of 53 MTAs (on all the 11 linkage groups) for all the four traits (Tables 5–9 and Figures 4, 5). The number of significantly associated markers for an individual trait varied from 9 for 100-seed weight to 18 for yield, with an average of 13.25 MTAs/trait. However, several common markers were found to be associated with more than one trait, and therefore the total unique MTAs discovered were 39 for all the four traits (Tables 5–9). A set of seven markers was such that influence more than one trait, i.e., these markers influence two to four traits (Table 9).

**TABLE 5:** Marker–trait associations (MTAs) identified for 100-seed weight in two different environments using GLM and MLM approaches of the software program TASSEL.

	Environment-I (Jammu)				Environment-II (Bhaderwah)			
Marker	Chromosome	*P*-value	PVE (%)	Model of detection	*P*-value	PVE (%)	Model of detection	Nature of MTA
BM140	4	0.005–0.017	13.1–18.6	GLM, MLM	0.015–0.033	11.7–16.4	GLM, MLM	Stable
BM154	9	0.02–0.04	11.2–14.1	GLM, MLM	0.02	10.6	GLM	Stable and already reported by Blair and Izquierdo (2012). The marker is also one of the flanking markers for seed weight QTL “Sw9.2”
BM164	2	0.0003–0.01	23.4–30.9	GLM, MLM	0.0004–0.01	22.9–29.5	GLM, MLM	Stable and major
BM199	4	0.001–0.01	14.1–18.6	GLM, MLM	0.01	10.8	GLM	Stable
BMb96	10	0.04	6.15	GLM	0.03–0.04	6.7–9.3	GLM, MLM	Stable
BMd25	8	0.0001–0.001	16.7–24.2	GLM, MLM	0.0003–0.002	15.8–22.8	GLM, MLM	Stable and major
BMR048	4	0.0004–0.004	16.4–23.4	GLM, MLM	0.001–0.008	14.8–20.7	GLM, MLM	Stable and major
BM172	3	0.01	13.2	GLM	–	–	–	
BM160	7	0.02	14.1	GLM	–	–	–	Already reported to be associated with DM, EP, PP, SP, and SPL by Galeano et al. (2012)

*GLM, general linear model; MLM, mixed linear model; PVE, phenotypic variation explained; DM, days to maturity; PP, pods per plant; SP, seed per pod; SPL, seed per plant; EP, empty pod%.*

**TABLE 6:** Marker–trait associations (MTAs) identified for seeds per pod in two different environments using GLM and MLM approaches of the software program TASSEL.

	Environment-I (Jammu)				Environment-II (Bhaderwah)			
Marker	Chromosome	*P*-value	PVE (%)	Model of detection	P-value	PVE (%)	Model of detection	Nature of MTA
BM172	3	0.003–0.03	21.9–24.9	GLM, MLM	0.03–0.04	17.8–21.1	GLM, MLM	Stable and major
BMR244	8	0.004–0.02	28.8–29.6	GLM, MLM	0.006–0.04	25.1–27.5	GLM, MLM	Stable and major
BMR269	8	0.02–0.03	11.2–11.7	GLM, MLM	0.002–0.006	14.7–16.1	GLM, MLM	Stable
Pvest008	2	0.02–0.03	20.5–24.3	GLM, MLM	0.0008–0.004	25.4–30.0	GLM, MLM	Stable and major
BMd02	2	0.02	11.5	GLM	0.003–0.02	13.3–14.1	GLM, MLM	Stable
BMd20	5	0.04	9.8	GLM	0.01–0.03	11.1–11.3	GLM, MLM	Stable and already reported by Blair and Izquierdo (2012) linked with seed weight QTL “Sw5.1”
BM160	7	0.03	25.9	GLM	–	–	–	Major and already reported to be associated with DM, EP, PP, SP, and SPL by Galeano et al. (2012)
BM210	7	0.008	18.7	GLM	–	–	–	Already reported to be associated with yield QTL by Asfaw et al. (2012)
BMd45	1	0.006–0.009	14.3–15.3	GLM, MLM	–	–	–	
PVBR113	6	0.01–0.02	15.8–17.6	GLM, MLM	–	–	–	
PVBR94	9	0.02	19.1	GLM	–	–	–	
BM150	7	–	–	–	0.01–0.05	12. 3–12.5	GLM, MLM	
Pvest258	4	–	–	–	0.001–0.007	21.8–24.2	GLM, MLM	Major
PVBR83	3	–	–	–	0.02	22.7	GLM	Major

*DM, days to maturity; PP, pods per plant; SP, seed per pod; SPL, seed per plant; EP, empty pod%.*

**TABLE 7:** Marker–trait associations (MTAs) identified for pods per plant in two different environments using GLM and MLM approaches of the software program TASSEL.

	Environment-I (Jammu)				Environment-II (Bhaderwah)			
Marker	Chromosome	*P*-value	PVE (%)	Model of detection	*P*-value	PVE (%)	Model of detection	Nature of MTA
BMd28	5	0.009–0.02	14.9–15.9	GLM, MLM	0.04–0.05	10.8–13.3	GLM, MLM	Stable and already reported to be associated with yield QTL “Yld5.1” by Blair and Izquierdo (2012)
BMR269	8	0.05	9.99	MLM	0.03–0.04	8.83–10.77	GLM, MLM	Stable
Pvm021	11	0.02	30.2	GLM	0.03	27.1	GLM	Stable and major
BM137	6	0.03	21.9	GLM	–	–	–	Major
BM151	8	0.01–0.04	17.76–18.82	GLM, MLM	–	–	–	
BM160	7	0.01	25.27	GLM	–	–	–	Major and already reported to be associated with DM, EP, PP, SP, and SPL by Galeano et al. (2012)
BM185	7	0.01–0.02	14.1–15.4	GLM, MLM	–	–	–	
BMd45	1	0.03–0.04	9.1–10.3	GLM, MLM	–	–	–	
BM172	3	–	–	–	0.03	16.3	GLM	
BMb96	10	–	–	–	0.04	8.7	GLM	
BMd01	3	–	–	–	0.03–0.04	7.4–8.8	GLM, MLM	

*DM, days to maturity; PP, pods per plant; SP, seed per pod; SPL, seed per plant; EP, empty pod%.*

**TABLE 8:** Marker–trait associations (MTAs) identified for yield per plant in two different environments using GLM and MLM approaches of the software program TASSEL.

	Environment-I (Jammu)				Environment-II (Bhaderwah)			
Marker	Chromosome	*P*-value	PVE (%)	Model of detection	*P*-value	PVE (%)	Model of detection	Nature of MTA
BM160	7	0.001	46.1	MLM	0.000007–0.003	41.3–43.5	GLM, MLM	Stable and major and already reported to be associated with DM, EP, PP, SP, and SPL by Galeano et al. (2012)
BM164	2	0.001	32.3	GLM	–	–	–	Major
BM172	3	0.00000006–0.001	34.5–41.5	GLM, MLM	0.000004–0.002	32.8–37.3	GLM, MLM	Stable and major
BM184	11	0.04	14.1	MLM	0.05	12.5	GLM	Stable
BM187	6	0.00002–0.03	30.9–39.5	GLM, MLM	0.0006–0.02	31.5–35.4	GLM, MLM	Stable and major
BMb96	10	0.00002–0.01	13.31–22.6	GLM, MLM	0.0001–0.01	14.9–21.6	GLM, MLM	Stable and major
BMd19	11	0.01	17.49	GLM	0.01	18.35	GLM	Stable and already reported to be associated with seed weight through SMA by Blair and Izquierdo (2012)
BMd41	11	0.002–0.02	17.66–19.7	GLM, MLM	0.004–0.02	18.9–19.8	GLM, MLM	Stable
BMd45	1	0.03–0.04	9.9–11.1	GLM, MLM	0.04–0.05	9.9–11.1	GLM, MLM	Stable
BMR269	8	0.008–0.02	10.4–15.5	GLM, MLM	0.007–0.02	11.3–16.1	GLM, MLM	Stable
Pvest006	2	0.0006–0.009	19.4–21.2	GLM, MLM	0.005–0.01	17.6–17.9	GLM, MLM	Stable
Pvest042	3	0.00000002–0.001	24.4–36.8	GLM, MLM	0.000006–0.002	23.4–31.7	GLM, MLM	Stable and major
Pvest072	6	0.04	27.1	GLM	0.02–0.04	30.8–34.2	GLM, MLM	Stable and major
PVBR251	2	0.01–0.03	16.5–17.1	GLM, MLM	–	–	–	
PVctt001	4	0.04	19.6	MLM	–	–	–	
PVBR112	4	–	–	–	0.02	15.9	GLM	
Pvest030	2	–	–	–	0.01–0.05	14.1–16.3	GLM, MLM	
BM154	9	–	–	–	0.04	14.6	GLM	Already reported to be associated with one of the flanking markers for seed weight QTL “Sw9.2” by Blair and Izquierdo (2012)

*DM, days to maturity; PP, pods per plant; SP, seed per pod; SPL, seed per plant; EP, empty pod%.*

**TABLE 9:** List of co-localized markers/QTLs associated with more than one trait.

Marker	Chromosome	Traits			
BM154	9	100SW	Yield		
BM160	7	100SW	PPP	SPP	Yield
BM164	2	100SW	Yield		
BM172	3	100SW	PPP	SPP	Yield
BMb96	10	100SW	PPP	Yield	
BMd45	1	PPP	SPP	Yield	
BMR269	8	PPP	SPP	Yield	

*PPP, pods per plant; SPP, seed per pod; SW, seed weight.*

For 100-seed weight, out of the nine MTAs (identified on LG02, 03, 04, 07, 08, 09, 10), four MTAs were declared stable (i.e., identified in both environments), and three MTAs were declared stable and major (i.e., identified in both environments and explaining >20% phenotypic variation for 100-seed weight). Among the nine MTAs, six MTAs were identified by both GLM and MLM, whereas three MTAs were identified by only one model, i.e., GLM (Table 5).

For seeds per pod, among the 14 MTAs (identified on LG01–LG09), three MTAs were declared stable, three MTAs were major, and three MTAs were declared both stable and major (i.e., identified in both environments and explaining >20% phenotypic variation for seeds per pod). Among the 14 MTAs identified for seeds per pod, 10 MTAs were identified by both GLM and MLM, whereas four MTAs were identified by only one model, i.e., GLM (Table 6).

For pod per plant, among the 11 MTAs (on LG01, 03, 05, 06, 07, 08, 10, 11), two MTAs were declared stable (i.e., identified in both environments), two MTAs are major (i.e., explaining >20% phenotypic variation for pod per plant), and one MTA is declared both stable and major. Six MTAs were identified by both GLM and MLM, whereas five MTAs were identified by only one model, i.e., GLM (Table 7).

For yield per plant, among the 18 MTAs (identified on all the linkage groups except LG05), six MTAs were declared stable, one MTA major, and six MTAs both stable and major. Among the 18 MTAs identified for yield per plant, 12 MTAs were identified by both GLM and MLM (Table 8).

It is important to note that 10 MTAs for all the four traits identified during the present study have also been found to be associated with grain yield or yield-contributing traits in earlier studies. Therefore, these 10 MTAs are declared as validated MTAs (Tables 5–9). The validated, major, and stable MTAs are considered important and will be recommended for common bean molecular breeding programs aimed at enhancing yield and yield-contributing traits.

## Discussion

Common bean (*P. vulgaris* L.) is one of the important grain legume crops for food and nutritional security in the world. The beans grown in the Himalayan region of Jammu and Kashmir, India possess huge diversity, and sometimes this region in India is considered as the secondary center of diversity for common bean. Common bean germplasm (landraces) grown in this Himalayan region possess huge diversity for seed color, shape, size, and flavor (Choudhary et al., 2018b). The insight on the origin and evolution of common bean germplasm grown in this region has been discussed by us in detail in an earlier study (Choudhary et al., 2018b). The study led to the conclusion that both gene pool species of common bean, i.e., Mesoamerican and Andean beans, are grown in the state of Jammu and Kashmir with the prevalence of Mesoamerican beans in the Jammu region and both Mesoamerican and Andean beans in the Kashmir region. These findings indicated multiple introductions of this crop in the hilly state of western Himalayas by travelers from different countries in the Indian subcontinent for trading in the early part of the 16th century *via* the Red and Arabian Sea and by Chinese travelers through the Hindustan Silk Route (Choudhary et al., 2018b). However, there is hardly any report available where this huge diversity has been characterized using sophisticated genomics tools and techniques and trait phenotyping in the field. For instance, earlier studies using germplasm from this region used morphological traits only for characterization (Sofi et al., 2014; Saba et al., 2016) or utilized less reliable RAPD markers (Zargar et al., 2016). In addition, these earlier studies used a very small collection of germplasm from only few hotspot regions. These limitations have been overcome in this study by using very precise genotypic platform (ABI 3730 automatic DNA Sequencer Genotyping Platform; Applied Biosystems, Foster City, CA, United States) using a diverse bean germplasm collection that represented all (11) hotspot regions in Jammu and Kashmir. In addition, exotic bean germplasm from 11 different countries were also included in the preset study. The results of trait analyses revealed desirable values of genetic parameters in the present core set of 96 common bean genotypes. The substantial variability available may provide opportunity to favorably improve yield and related traits through selection. The elucidation of variability in the population is of paramount importance to frame an appropriate breeding strategy for seeking improvement of economically important traits. However, it is very important to mention here that yield is a very complex quantitative trait that is controlled by a network of large number of small effect minor genes/QTLs. The detection of these small effect genes/QTLs may escape detection in a small population using less number of markers. Therefore, there is a scope of using large populations/large germplasm collections with more number of markers in the future to capture more number of small effect minor genes/QTLs. Nevertheless, this study provided a promising insight for the first time into the complex genetic architecture of grain yield in different environments of western Himalayas, and findings may prove useful for common bean improvement programs worldwide.

### Germplasm Characterization, Genetic Diversity, and Population Structure Analyses

The study of allelic diversity using all the 91 SSR markers on a diverse set of 96 lines revealed a very high diversity in the common bean germplasm from the state of Jammu and Kashmir, India. This is evident by the detection of up to 21 alleles by SSR marker BM187, very high average number of alleles/locus (7.59), and high average gene diversity (*He* = 0.73) (Table 3 and Supplementary Table 2). The results are very encouraging and may be partly due to the precise ABI sequencing system used for SSR genotyping during the present study. The results also supported the belief that common bean germplasm being grown in north-western Himalayas is very diverse and can be used in gene discovery programs and genetic improvement of common bean. The comparison with few earlier studies revealed that the diversity in our common bean germplasm is more than the Chinese common bean germplasm (Zhang et al., 2008), USDA common bean core collection (McClean et al., 2012), and Portuguese common bean germplasm (Leitao et al., 2017).

The high diversity of common bean from this region can also be predicted by the fact that the local landraces were almost as diverse as exotic common bean germplasm used in the present study. The local landraces got uniformly distributed along with exotic lines during cluster analysis (ESM Figure 1). Little difference has been noticed in allelic diversity: the local landraces possess 6.7 avg. no. of alleles/locus against 6.82 avg. no. of alleles/locus in exotic germplasm. Similarly, little difference has been noticed for the number of private alleles, the number of alleles with a frequency ≥ 5%, and gene diversity values between exotic and local landraces of common bean from the state of Jammu and Kashmir (Table 3).

In our present study, we noticed that the common bean germplasm from the Andean gene pool possess more diversity than the germplasm from the Mesoamerican gene pool. For instance, more total number of alleles and average number of alleles were detected in common bean germplasm belonging to the Andean gene pool than the germplasm belonging to the Mesoamerican gene pool (Table 3). However, in earlier studies, an opposite trend, i.e., more number of alleles using genic and genomic SSR markers, has been shown in Mesoamerican beans than in Andean beans (Zhang et al., 2008). These results obtained during the present study may be partly due to more number of private alleles detected in Andean beans (1.29) than in Mesoamerican beans (1.25). The greater diversity in Andean beans than in Mesoamerican beans is considered a feature of SSR marker analysis, and these results got support from some earlier studies (Blair et al., 2006; Zhang et al., 2008; McClean et al., 2012). The gene diversity trends showed that Mesoamerican beans are more diverse (0.67) than Andean beans (0.65). Similar results have been reported earlier as well using isozymes (Koenig and Gepts, 1989), RFLP (Becerra-Velasquez and Gepts, 1994), RAPD (Beebe et al., 2000, 2001), AFLP (Tohme et al., 1996), and DNA sequence data (McClean et al., 2004; McClean and Lee, 2007).

We also observed that the genic markers reveal less diversity than random SSR markers, as has been reported in several earlier studies (Blair et al., 2006; Zhang et al., 2008). However, the diversity revealed by genic markers reflects true diversity of a crop species.

The diverse nature of germplasm collection used during the present study was also evident by the fact that all the 96 lines were clustered uniformly and do not form any specific cluster for local landraces and exotic lines (ESM Figure 1). On the other hand, there was clear-cut assignment/clustering of lines based on their phaseolin patterns with the clustering of Andean types separately from the Mesoamerican types in both traditional hierarchical clustering and Bayesian clustering through structural analysis (Figures 2A,B). Similar results (only two subpopulations) have also been reported in population structural analysis in earlier studies (Zhang et al., 2008; Leitao et al., 2017), and the two subpopulations corresponded to the Andean and Mesoamerican gene pools (Zhang et al., 2008; Kwak and Gepts, 2009; McClean et al., 2012). The presence of only two subpopulations in the Himalayan beans is typical to most legume crops due to the self-pollinating nature of the legume crops. In summary, both distance and model-based approaches classified our common bean collection into two major subpopulations, and these results are consistent with previous results that recognized two major subdivisions within the cultivated common bean (Gepts et al., 1986; Singh et al., 1991; Becerra-Velasquez and Gepts, 1994; Kwak and Gepts, 2009; McClean et al., 2012).

The information of structure will be useful to avoid spurious association in the study of MTAs through GWAS. The results of structural analysis and UPGMA clustering are in agreement since in both the clustering types, two distinct groups were formed based on two different gene pools, i.e., Mesoamerican vs. Andean gene pools (Leitao et al., 2017).

### Gene Discovery for Yield and Yield-Contributing Traits

In common bean, significant and positive correlations were observed between yield and its component traits including 100-seed weight, pods per plant, and seeds per pod during the present study and in some earlier studies as well (Beebe et al., 2013; Assefa et al., 2015, 2019; Rao et al., 2017). Therefore, yield components could be used as selection criteria for the improvement of yield and the development of next-generation common bean cultivars. In fact, it is well documented that an increase in yield in common bean under favorable environmental conditions has come from improvement in pods per plant, seed per plant, and 100-seed weight (Beebe et al., 2013; for review, see Assefa et al., 2019).

During the present study, a set of 39 significantly associated markers/genes on all the 11 chromosomes has been identified for all the four traits. This includes 15 major MTAs, 15 stable MTAs, and 13 both major and stable MTAs. One of the most important breakthroughs achieved during the present study is the validation of a set of 10 MTAs already identified in earlier studies. Some of the validated markers found correspondence to some important QTLs for yield and yield-contributing traits (Tables 5–9). For instance, SSR marker “BM154” associated with 100-seed weight and yield on chromosome 9 has also been reported in an earlier study by Blair and Izquierdo (2012) for seed weight. The marker “BM154” is one of the associated flanking markers for the seed weight QTL “Sw9.2.” The marker “BMd20” found to be associated with trait “seeds per pod” during the present study has been earlier identified and found to be linked with seed weight QTL “Sw5.1” (Blair and Izquierdo, 2012). The important marker “BM160” found to be associated with all the four traits (pods per plant, seeds per pod, 100-seed weight, and yield per plant) during the present study has also been found to be associated with a variety of yield-related traits (days to maturity, pods per plant, seed per pod, seed per plant, and empty pod%) in an earlier study (Galeano et al., 2012). The marker “BM210” identified to be associated with seeds per pod during the present study has been found to be associated with yield by Asfaw et al. (2012). Stable QTL-linked marker “BMd28” identified during the present study for “pods per plant” has been already reported to be associated with yield QTL “Yld5.1” by Blair and Izquierdo (2012). Similarly, marker “BMd19” found to be associated with yield per plant during the present study has been found to be associated with seed weight through single marker analysis (Blair and Izquierdo, 2012). The major, stable, and validated MTAs for yield and yield-contributing traits may be used in common bean breeding programs aimed at enhancing yield of common bean.

In common bean, different trait mapping studies have been already conducted using both bi-parental mapping populations and more recent GWASs involving diverse germplasm collections (for review, see González et al., 2018; Assefa et al., 2019). In these earlier studies, several genes with minor effects involved in the genetic control of seed size, pod size, and yield have been identified repeatedly in different genetic backgrounds with increasingly tight genetic bounds (González et al., 2018; Assefa et al., 2019). For instance, genes for pod size and pod length have been identified in some earlier studies at similar locations on LG01, LG02, and LG04 (Koinange et al., 1996; Yuste-Lisbona et al., 2014; Hagerty et al., 2016). In another study using single-point analysis, a set of 10-positive markers was found to be associated with yield on linkage groups b01, b02, b03, b04, and b09, and 21 markers were found to be associated with seed size (Blair and Izquierdo, 2012). Using composite interval mapping, nine markers were identified for seed weight across four linkage groups (b02, b03, b05, and b09), and one QTL was detected for yield on linkage group b05 (Blair and Izquierdo, 2012). Significant MTAs have also been identified for other yield components including pods per plant (PP), seed per pod (SP), and seed per plant (SPL) through association mapping (Galeano et al., 2012). A number of common bean genes/QTLs for yield and yield-contributing traits have been projected on all the 11 linkage groups except linkage group 01 (LG01) of the consensus reference genetic map developed from genetic maps of three populations^5^. The total number of QTLs for yield and yield-contributing traits projected on 10 linkage groups (LG02 to LG11) is 85 and varies from three QTLs (LG05 and LG11) to 21 QTLs (LG06) with an average of 8.5 QTLs/linkage group. The co-localized markers that influence more than one trait will prove useful in the simultaneous improvement of multiple traits in common bean. The markers BM160 and BM172 that influence all the four traits (pods per plant, seeds per pod, 100- seed weight, and yield per plant) are considered most important markers for breeding programs aimed at enhancing grain yields in common bean.

## Data Availability Statement

The datasets presented in this study can be found in online repositories. The names of the repository/repositories and accession number(s) can be found in the article/Supplementary Material.

## Author Contributions

RM conceived the idea, conducted the experiments, and wrote the manuscript. NC, VB, and SJ performed the experiments. BS helped in collection and analysis of field data. AK and MAB helped in manuscript writing and analysis of data. RP helped in analysis of data for GWAS. MT, AC, and RV helped in generation of genotypic data and data analysis. All authors contributed to the article and approved the submitted version.

## Conflict of Interest

The authors declare that the research was conducted in the absence of any commercial or financial relationships that could be construed as a potential conflict of interest.

## Footnotes

1 http://faostat.fao.org/faostat

2 https://windostat.software.informer.com/

3 http://www.indostat.org/

4 http://www.maizegenetics.net

5 https://legumeinfo.org/traits_maps/cmap/?ref_map_set_acc=PvConsensus_GaleanoFernandez2011_a&ref_map_accs=1
