# Supplementary material for: Allelic Diversity, Structural Analysis, and Genome-Wide Association Study (GWAS) for Yield and Related Traits Using Unexplored Common Bean (Phaseolus vulgaris L.) Germplasm From Western Himalayas
Source: Front Genet. 2021 Jan 28;11:609603. doi: 10.3389/fgene.2020.609603 (PMC7876396; doi:10.3389/fgene.2020.609603)
Supplement: Supplementary file 4 [file Table_4.DOCX]

**ESM Table 4: Phenotypic correlation coefficient of 96 common bean lines obtained using data recorded at SKUAST-Jammu and at Bhaderwah locations.**

| **Character** | **Seeds per pod** | **100 seed weight (g)** | **Yield per plant (g)** |
| --- | --- | --- | --- |
| **Pods per plant** | 0.1618**  *0.3003*** | -0.1735**  *-0.1884*** | 0.5645**  *0.6435*** |
| **Seeds per pod** | - | 0.0705  *-0.0873* | 0.4917**  *0.5081*** |
| **100 seed weight (g)** |  | - | 0.5359**  *0.4439*** |

*** -** significant at 5% level**, **-** significant at 1% level ; *italicized values are for Bhaderwah data*
