# Supplementary material for: Allelic Diversity, Structural Analysis, and Genome-Wide Association Study (GWAS) for Yield and Related Traits Using Unexplored Common Bean (Phaseolus vulgaris L.) Germplasm From Western Himalayas
Source: Front Genet. 2021 Jan 28;11:609603. doi: 10.3389/fgene.2020.609603 (PMC7876396; doi:10.3389/fgene.2020.609603)
Supplement: Supplementary file 5 [file Table_5.DOCX]

| **ESM Table 5: Mean LnP (K) and Delta K values of assumed sub-populations (K= 1 to 10) during structural analysis in 96 common bean lines** | | | | | | |
| --- | --- | --- | --- | --- | --- | --- |
| **K** | **Reps** | **Mean LnP(K)** | **Stdev LnP(K)** | **Ln'(K)** | **\|Ln''(K)\|** | **Delta K** |
| 1 | 3 | -5350.4 | 0.70238 | — | — | — |
| **2** | **3** | **-3954** | **0.75498** | **1396.43** | **1220.03** | **1615.97** |
| 3 | 3 | -3777.6 | 19.6695 | 176.4 | 244.1 | 12.4101 |
| 4 | 3 | -3357.1 | 35.3378 | 420.5 | 442.267 | 12.5154 |
| 5 | 3 | -3378.9 | 139.815 | -21.767 | 238.3 | 1.7044 |
| 6 | 3 | -3162.3 | 84.51 | 216.533 | 171.533 | 2.02974 |
| 7 | 3 | -3117.3 | 183.742 | 45 | 33.6 | 0.18287 |
| 8 | 3 | -3105.9 | 310.217 | 11.4 | 201.7 | 0.65019 |
| 9 | 3 | -2892.8 | 100.826 | 213.1 | 377.267 | 3.74177 |
| 10 | 3 | -3057 | 169.918 | -164.17 | — | — |
