# Supplementary figures and images for: Allelic Diversity, Structural Analysis, and Genome-Wide Association Study (GWAS) for Yield and Related Traits Using Unexplored Common Bean (Phaseolus vulgaris L.) Germplasm From Western Himalayas
Source: Front Genet. 2021 Jan 28;11:609603. doi: 10.3389/fgene.2020.609603 (PMC7876396; doi:10.3389/fgene.2020.609603)

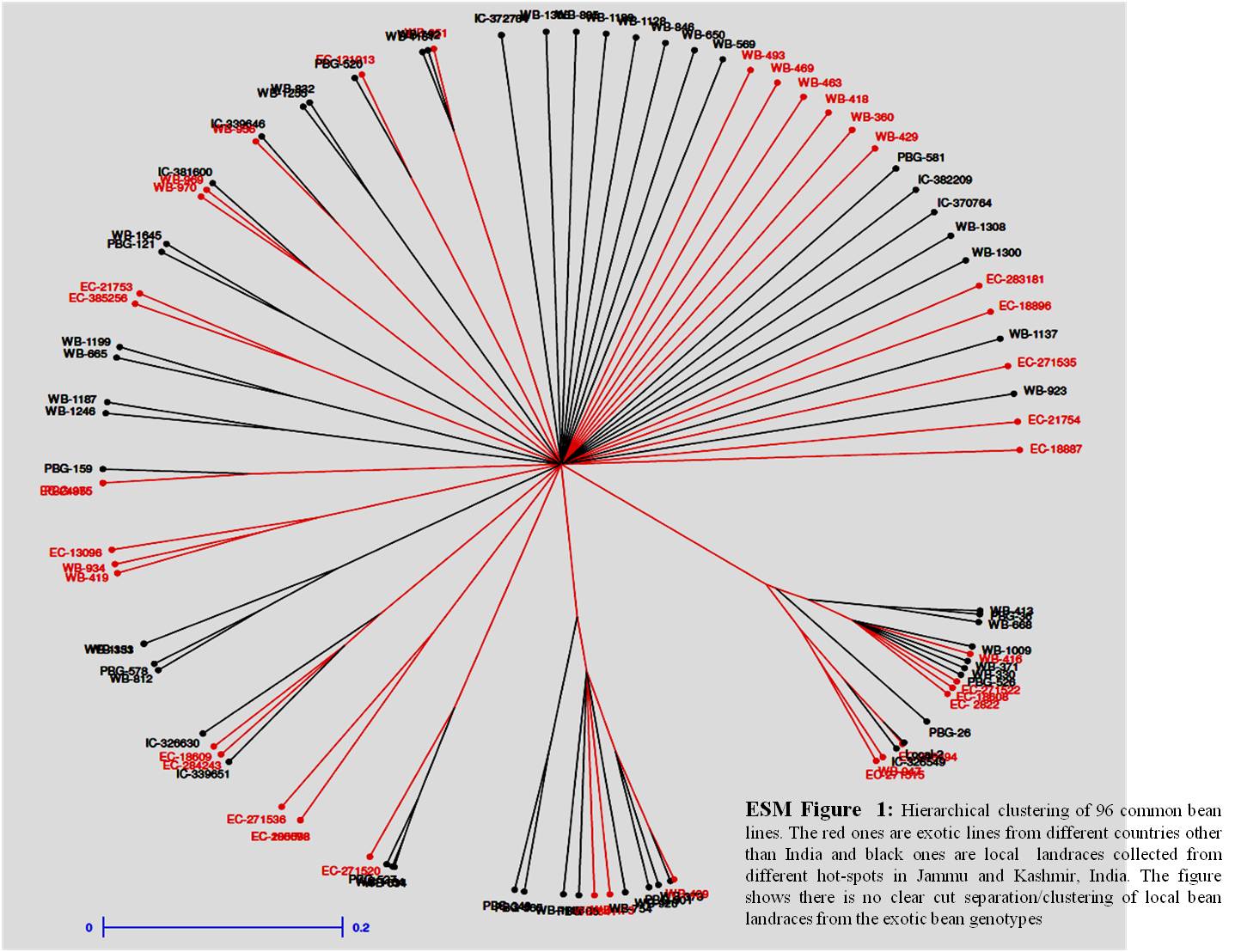

Supplement: Supplementary file 6 [file Image_1.JPEG]
